# Posterior Canal and Atypical Benign Paroxysmal Positional Vertigo: Development of a Predictive Model for Clinical Decision-Making

**DOI:** 10.7759/cureus.111292

**Published:** 2026-06-22

**Authors:** Kathrine M Jakobsen, Line M Nielsen, Clara Bender, Simon L Cichosz, Michelle R Petrak, Sara-Sofie S Kirkeby, Amy R Cassidy, Susan L Whitney

**Affiliations:** 1 Department of Health Science and Technology, Aalborg University, Aalborg, DNK; 2 Department of Audiology, Interacoustics, Middelfart, DNK; 3 Department of Software Development, Interacoustics, Middelfart, DNK; 4 Department of Physical Therapy, University of Pittsburgh Medical Center Rehabilitation Institute, Pittsburgh, USA

**Keywords:** bppv, clinical decision support, diagnosis, latency, predictive modeling, slow-phase velocity

## Abstract

Background: Benign paroxysmal positional vertigo (BPPV) is the most common cause of dizziness. Despite its prevalence, clinical diagnosis can be challenging because of difficulty discerning the direction and intensity of eye movements, resulting in diagnostic uncertainty and suboptimal treatment outcomes. The aim of this study was to develop and evaluate a data-driven model designed to differentiate between typical and atypical variants of BPPV.

Methods: A retrospective cross-sectional design included data from 26 patients diagnosed clinically with either typical BPPV or atypical variants of BPPV and two control subjects with a history of dizziness but no vertigo. Eye and head movement data were extracted using VisualEyes™ (Interacoustics, Middelfart, Denmark) and processed in MATLAB (MathWorks, Natick, Massachusetts, United States). Latency and peak slow-phase velocity (SPV) of torsional nystagmus were used as predictors in a logistic regression model. Model performance was evaluated using receiver operating characteristic (ROC) curve analysis, area under the ROC curve (AUC), and repeated five-fold cross-validation.

Results: The final model, based on latency and peak SPV of torsional nystagmus, achieved a mean AUC of 0.936±0.007, indicating excellent discrimination between typical and atypical BPPV. Across cross-validation folds, the model demonstrated a mean accuracy of 88.0%±0.9%, a sensitivity of 78.2%±1.9%, and a specificity of 93.9%±1.1%, reflecting a consistently high rate of correct classification. When evaluated on four previously unseen patient recordings outside the training dataset, the model showed 100% agreement with expert clinical assessments.

Conclusion: The developed model demonstrates high diagnostic accuracy in differentiating typical BPPV from atypical variants and shows promise as a clinical decision support tool.

## Introduction

Benign paroxysmal positional vertigo (BPPV) is the most common vestibular disorder, accounting for up to 42% of all dizziness cases encountered in clinical settings [1,2]. BPPV is often associated with recurrent vertigo that is provoked by changes in head position relative to gravity and typically lasts up to one minute [3,4]. The lifetime prevalence of BPPV has been estimated at 2.4% based on a telephone survey conducted in Germany, with approximately 1.1 million adults experiencing BPPV annually. The cause of BPPV is most commonly idiopathic; however, it may also be associated with underlying pathologies such as head trauma, vestibular neuritis, and migraine. Although BPPV is termed "benign" in that it is not life-threatening, its impact on quality of life, fall risk, and healthcare utilization is far from negligible [5,6]. Agrawal et al. [7] reported that chronic vestibular dysfunction results in an average loss of 1.30 quality-adjusted life years per affected patient, corresponding to approximately $64,929 in lost economic value per patient over a lifetime. When extrapolated to the broader older adult population in the United States, this equates to an estimated $227 billion in societal costs related to reduced quality of life and productivity [7]. Thus, while BPPV is labeled "benign" in terms of mortality, it carries substantial socioeconomic and personal burdens.

BPPV typically manifests in what clinicians consider the "typical BPPV" form, which refers to canalithiasis in the posterior semicircular canal. However, it can also present in a variety of atypical variants. For the purposes of this study, we define typical BPPV as the classic posterior canal canalithiasis type, with its characteristic positional nystagmus of upbeating geotropic torsional nystagmus (beating towards the earth) in the Dix-Hallpike position [3,4]. Typically, BPPV is associated with the perception of vertigo/dizziness that fatigues within one minute and is associated with a change of head position relative to gravity and with torsional upbeating nystagmus with a typical crescendo-decrescendo pattern that changes direction after returning from the Dix-Hallpike position. Correspondingly, we define atypical BPPV as any other variant of BPPV that does not meet the criteria of the classic posterior canal canalithiasis. Atypical BPPV, therefore, encompasses BPPV involving other canals, including horizontal and anterior canal BPPV, as well as less common presentations such as cupulolithiasis or multiple-canal involvement [3,4]. Distinguishing between typical BPPV and these atypical variants is clinically important because the choice of treatment maneuver depends on the affected canal.

Posterior canal BPPV is the most common subtype of BPPV, accounting for approximately 80-90% of all BPPV cases, and is typically identified by the characteristic torsional upbeating nystagmus elicited during the Dix-Hallpike maneuver [2]. However, several less common or atypical variants of posterior canal BPPV have been described, often presenting with altered nystagmus patterns that may complicate the identification of the affected canal and the appropriate therapeutic maneuver [8]. Hence, there is a need for an algorithm that can detect typical from atypical posterior canal BPPV. Santopietro et al. [8] reported that the Scocco variant of posterior canal BPPV, where patients report vertigo only upon sitting up, was more resistant to repositioning maneuvers, while in the Dix-Hallpike position, little to no nystagmus has been reported with the Scocco posterior canal variant [9]. Identification of these less typical posterior canal BPPV patterns may help to estimate the time to recovery with repositioning maneuvers.

Rasmussen et al. [10] and Martens et al. [11] reported positional nystagmus using video goggles in 70-88% of healthy subjects when tested in a mechanical chair and during the standard Dix-Hallpike. With the use of video goggles, clinicians are "seeing" nystagmus that may be normal, which makes diagnosing posterior canal BPPV more complex.

Diagnosis of BPPV relies on observing the characteristic nystagmus elicited by positional tests such as the Dix-Hallpike Test (DH-T) or supine roll test and comparing these findings with the patient's reported symptoms [3,12,13]. However, in some cases, the nystagmus responses can be subtle, ambiguous, or even absent, leading to diagnostic uncertainty. Factors such as examiner experience and subjective interpretation play a significant role in traditional BPPV diagnosis [3].

In recent years, advanced diagnostic tools have been introduced to aid in the diagnostic process. Video nystagmography (VNG) goggles and similar devices can objectively record eye movements during positional tests, and inertial sensors can track head movements [10]. These tools provide quantifiable, objective measurements of nystagmus and allow reproducible testing that is less reliant on an individual examiner's observations. Studies have shown that VNG-based positional testing can detect BPPV with sensitivity comparable to traditional bedside examination methods without increasing false-positive diagnoses, suggesting that such technology can augment clinical accuracy [10]. However, the clinical utility of these advanced diagnostic technologies ultimately hinges on the proper interpretation of the data. The clinician's expertise in recognizing the pattern of nystagmus and correlating it with the affected canal remains essential to distinguishing BPPV from other causes of vertigo and to determining the appropriate management. Advances in diagnostic tools combined with clinical experience may assist in making the diagnosis of BPPV, thereby improving patient outcomes and reducing the socioeconomic burden of BPPV [6].

The identification of the involved canal and the appropriate therapeutic maneuver can be complex based on atypical presentations of BPPV [14]. Diagnostic accuracy based solely on clinical features may be limited, with reported classification accuracies of approximately 60-80% across BPPV subtypes [15]. Furthermore, the Dix-Hallpike maneuver itself has a reported sensitivity of approximately 79% [16], indicating that some cases may be missed or misinterpreted during bedside examination. Objective quantification of nystagmus characteristics using video oculography may therefore assist clinicians in distinguishing typical from atypical positional nystagmus patterns and support more accurate canal identification and treatment selection.

Recently, computational approaches have been explored to assist in the analysis of eye movement recordings in vestibular diagnostics. Several studies have investigated the automated detection of nystagmus using machine learning techniques applied to video oculography data. Convolutional neural network (CNN) models have achieved classification performance exceeding 80-90% [17,18]. Moreover, deep learning approaches have been developed to identify torsional components of nystagmus from infrared recordings, facilitating the automated detection of the affected semicircular canal in BPPV [19]. These studies demonstrate the growing interest in applying computational methods to vestibular diagnostics, although most focus on detecting the presence of nystagmus rather than quantitatively characterizing temporal response features during positional testing.

It may be possible to leverage data derived from video goggles to assist in clinical decision-making. A data-driven model that analyzes quantitative nystagmus features may support clinicians by providing an evidence-based prediction of whether a given presentation is a form of typical posterior canal BPPV or an atypical variant. Therefore, the purpose of this study was to develop and internally validate a predictive model capable of differentiating typical BPPV vs. atypical variants of BPPV using objective features obtained during the DH-T. We hypothesized that the dynamic characteristics of nystagmus contain sufficient information to distinguish typical posterior canal BPPV from atypical presentations and that a logistic regression model based on these features could achieve clinically useful accuracy.

## Materials and methods

Study design

The project was an observational cross-sectional study in diagnostic modelling, with a retrospective approach [20]. The study was conducted at the Department of Physical Therapy of the University of Pittsburgh Medical Center Rehabilitation Institute in Pittsburgh, Pennsylvania, after obtaining approval from the University of Pittsburgh Institutional Review Board (approval number: STUDY22020106). Existing clinical data from patient cases prospectively collected for an ongoing study were used without any intervention or randomization [1]. The diagnosis was determined by an expert clinician with 13 years of full-time experience working with people with vestibular disorders and 17 total years of experience as a physiotherapist, whose clinical decisions served as a reference during both training and testing of the model.

Data collection

**Figure 1:**
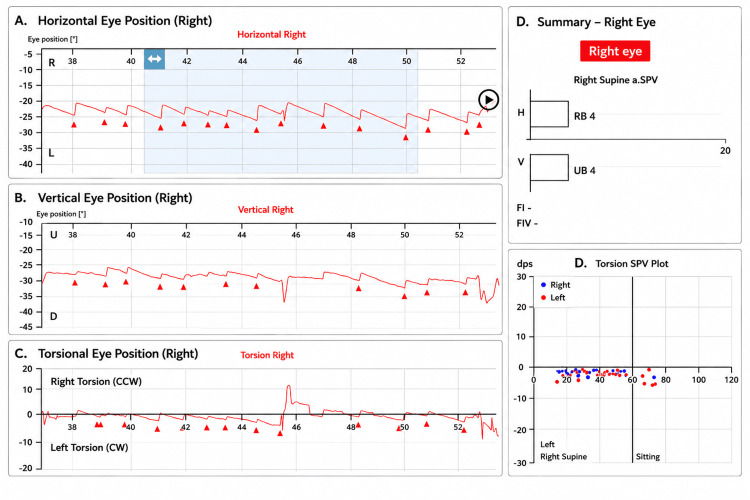
Dix-Hallpike recording demonstrating low-amplitude positional nystagmus with minimal torsional component (A) Horizontal eye position trace recorded during the right Dix-Hallpike maneuver. Horizontal nystagmus was present with a right-beating component. (B) Vertical eye position trace recorded during the right Dix-Hallpike maneuver. Vertical nystagmus demonstrated an upbeating component. (C) Torsional eye position trace recorded during the right Dix-Hallpike maneuver. Torsional eye movements were minimal throughout testing despite the presence of horizontal and vertical nystagmus components. (D) Summary of aSPV measurements and torsion SPV analysis. Horizontal and vertical aSPV values demonstrated right-beating and upbeating nystagmus, while torsion SPV measurements remained near baseline, indicating a negligible torsional component. aSPV: angular slow-phase velocity; SPV: slow-phase velocity; CCW: counterclockwise; CW: clockwise

The dataset was provided by the University of Pittsburgh Medical Center and included VNG recordings from 26 total patients, 24 of whom had BPPV symptoms of vertigo associated with a change of head position. All patients underwent a bilateral DH-T after eye calibration. Eye and head movements were recorded using VNG. Latency and peak slow-phase velocity (SPV) of torsional nystagmus were used as predictors in a logistic regression model. An atypical BPPV VNG trace for the right DH-T is illustrated in Figure 1. Deidentified data were shared via a secure Microsoft Teams channel in accordance with the data protection agreement and compliance with the General Data Protection Regulation (GDPR) and the Health Insurance Portability and Accountability Act (HIPAA).

Each patient underwent a diagnostic DH-T. Inclusion criteria for the dataset were patients with positional vertigo who had a confirmed BPPV diagnosis (typical/atypical) as determined by expert clinicians and two control subjects with no reports of vertigo. Because this was a retrospective analysis of an existing dataset, there were no specific prospective exclusion criteria beyond having incomplete data records. All BPPV cases had been classified by the treating vestibular specialist as either "typical BPPV" or an "atypical" form of BPPV based on clinical examination and positional test findings.

Table 1 provides additional demographic and clinical characteristics of the patients included in the analysis. These expert-confirmed clinical diagnoses were used as the ground-truth labels for the analysis, serving as the reference for model development. Typical BPPV cases were defined as presenting with posterior canal canalithiasis, whereas atypical BPPV cases included four patients with horizontal canal BPPV and two persons without BPPV. This classification was made independently of the data analysis and was based on the clinical diagnosis documented for each patient. 

**Table 1:** Demographic and clinical characteristics of patients with posterior canal BPPV (n=20), horizontal canal BPPV (n=4), and no symptoms of BPPV (n=2) Patient demographics, repositioning maneuvers, testing frequency, and self-reported dizziness severity are presented for all study participants. Dizziness severity was self-reported on a 10-point scale at presentation. BPPV: benign paroxysmal positional vertigo; F: female; M: male; L: left; R: right; N/A: not available

Subject	Classification	Repositioning maneuver	Age (years)	Tests (n)	Visits (n)	Sex	Dizziness severity
1	Typical	Left modified Epley	68	4	2	F	6/10 (L)
2	Typical	Left Gufoni	38	2	1	M	5/10 (L)
3	Typical	Left modified Epley	73	8	4	F	8/10 (L)
4	Typical	Left modified Epley	36	4	2	F	8/10 (L)
5	Typical	Left Gufoni + Li Quick Roll	61	1	1	M	2-4/10 (L)
6	Typical/atypical	Left modified Epley	68	2	1	F	3/10 (L)
7	Typical/atypical	Left modified Epley	54	4	2	F	7/10 (L)
8	Typical/atypical	Right modified Epley	65	2	1	F	10/10 (L)
9	Typical/atypical	Right Semont	71	1	1	M	10/10 (L)
10	Typical/atypical	Left modified Epley	59	7	4	F	8/10 (L)
11	Typical/atypical	Left modified Epley	74	6	3	M	4/10 (R); 6-7/10 (L)
12	Typical/atypical	Left Semont/Epley	65	23	8	F	N/A
13	Atypical	Left Semont	76	4	2	F	1-2/10 (R); 3-4/10 (L)
14	Atypical	Right modified Epley + Li posterior	57	4	2	F	10/10 (R); 1-2/10 (L)
15	Atypical	Right Demi Semont	57	4	2	F	3/10 (R)
16	Atypical	Right Demi Semont	89	4	2	F	5/10 (L)
17	Atypical	Left modified Epley	57	4	2	F	6/10 (L)
18	Atypical	Left Semont/Semont Plus	66	8	4	F	3/10 (R); 4/10 (L)
19	Atypical	Right modified Epley	74	6	3	F	7/10 (R); 3/10 (L)
20	Atypical	Right Gufoni	47	6	3	F	4/10 (R); 3/10 (L)
21	Atypical	Left Li Quick Roll	73	2	1	F	6/10 (L)
22	Atypical	Left Epley	74	6	3	F	7/10 (L); 5/10 (R)
23	Atypical	Left Li (posterior canal)	62	8	4	F	6-7/10 (L)
24	Atypical	Right modified Epley	79	6	3	F	5/10 (R)
25	Atypical	No maneuver	74	4	2	M	2/10 (R); 2/10 (L)
26	Atypical	No maneuver	72	3	2	F	1/10 (R); 2/10 (L)

Data processing and analysis workflow

**Figure 2:**
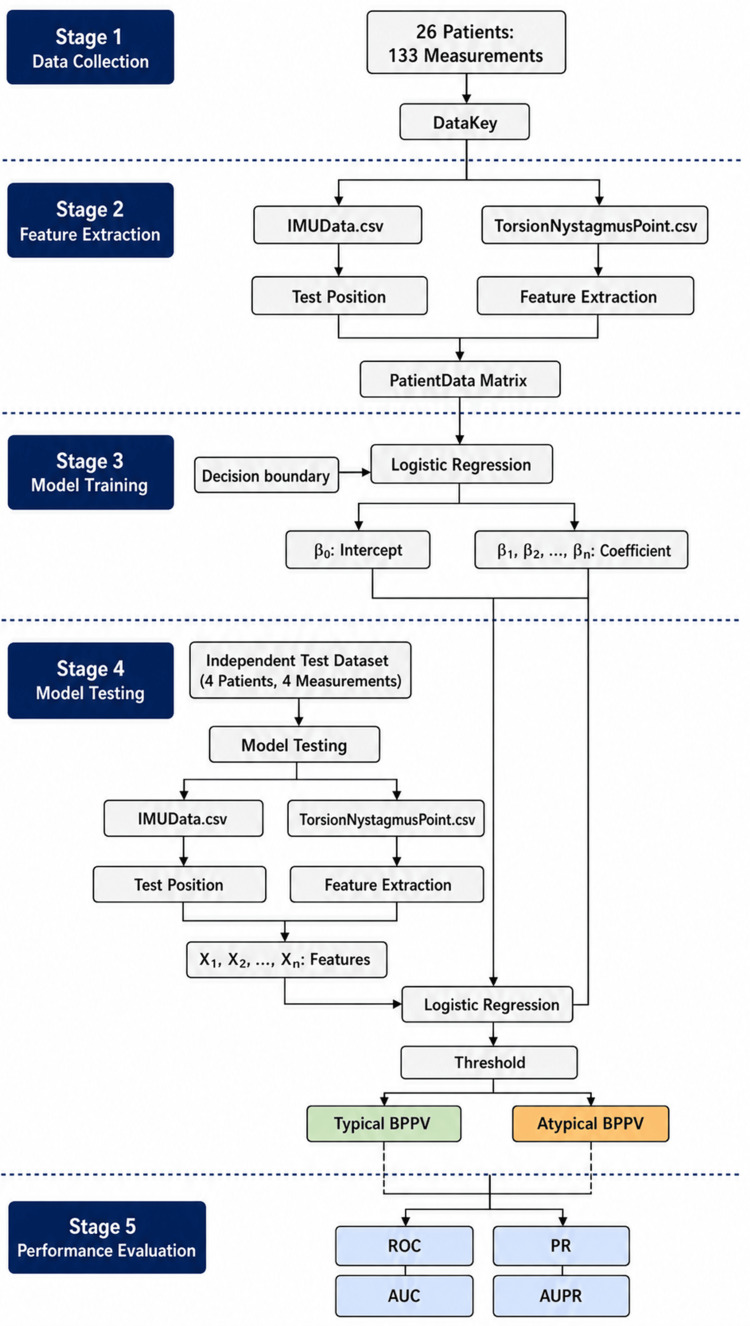
Machine learning workflow for the classification of typical and atypical BPPV Overview of the machine learning pipeline used to classify BPPV recordings as typical or atypical. Stage 1 involved data collection and the creation of a unified data key from 26 patients and 133 measurements. Stage 2 included data preprocessing, the extraction of IMU data and torsional nystagmus point data, the identification of test position, and the calculation of derived features to generate a patient data matrix. Stage 3 involved the development of a logistic regression model, including the determination of model coefficients and decision boundaries. Stage 4 evaluated model performance using an independent test dataset comprising four patients and four measurements. Extracted features (X1-Xn) were processed by the trained logistic regression model and classified according to a predefined threshold as typical BPPV or atypical BPPV. Stage 5 assessed classification performance using ROC curve and PR analyses. BPPV: benign paroxysmal positional vertigo; IMU: inertial measurement unit; ROC: receiver operating characteristic; AUC: area under the receiver operating characteristic curve; PR: precision-recall; AUPR: area under the precision-recall curve

The analytical workflow consisted of five stages: (1) data structuring, (2) preprocessing and feature extraction, (3) model development and internal validation, (4) clinical implementation, and (5) validation (Figure 2).

Stage 1: Structuring Raw Data

Each DH-T produced two datasets (one per eye), which were treated as independent observations. Repeated tests from the same patient were also included as separate entries. As BPPV often occurs unilaterally, an observation was defined as a single DH-T performed on one side, resulting in a total of 133 observations from the 26 patients.

Stage 2: Preprocessing and Feature Extraction

Vestibular data were recorded using VNG goggles (VORTEQ™, VisualEyes™, Interacoustics, Middelfart, Denmark) equipped with an integrated tri-axial inertial measurement unit (IMU). Two synchronized data streams were generated for each test: head motion and torsional eye movement. These were stored as paired files and indexed using a metadata key file ("DataKey") containing patient ID, test date, test side, eye, diagnostic label (1=typical BPPV; 0=atypical BPPV), and file paths.

Raw signals were smoothed using a moving average window of 0.2-0.3 s, adjusted according to the sampling rate (100-250 Hz). Feature extraction focused on clinically relevant parameters including latency, SPV, rise time, fall time, and total duration. Extracted features were stored in a structured matrix ("PatientData").

IMU-based identification of test position: VORTEQ™ IMU data were used to identify the precise moment of test position (transition from movement to stillness). Before analyzing nystagmus metrics, a time-reference point (Test Position) was established for each observation. This reference was defined as the moment the patient's head reached the position of the DH-T (supine with head rotated 45° to one side and extended ~20° below horizontal).

Objectively, this was determined by analyzing the first 10 seconds of IMU data to identify peak movement during the transition, establishing an individualized threshold at 20% of this peak, and defining Test Position as the first moment in a one-second sliding window when movement fell below this threshold.

The algorithm analyzed these signals to detect the end of the brisk deceleration phase as the moment when the head movement abruptly stops and remains stationary in the test position.

Test position was algorithmically identified as a shift in the IMU signal pattern, marking the end of active head motion. It served as a physiological and reproducible marker for the onset of the positional stimulus. All subsequent measurements of nystagmus, including latency and peak SPV, were time-aligned relative to this reference point. The test position served as an objective and physiologically relevant anchor that assured consistent temporal alignment across all trials and participants.

Latency was defined as the time from attaining the test position to the onset of the first observable torsional nystagmus (in seconds). This represents the delay before nystagmus begins after the positional change [19]: $\mathrm{Latency} (\mathrm{s})=\text{tfirst nystagmus}-\mathrm{ttestposition}$.

Peak SPV was the maximum SPV of the torsional nystagmus, measured in degrees per second (°/s). This is essentially the highest intensity (fastest eye movement) achieved by the nystagmus during the test [17,18].

Rise time was the time from the first detected nystagmus to the moment of peak SPV. This measures how quickly the nystagmus builds up to its maximum intensity (in seconds): $\text{Rise time}(\mathrm{s})=\text{tPeak SPV}-\text{tfirst nystagmus}$.

Fall time was the time from the moment of peak SPV to the end of nystagmus (last observed nystagmus beat). This measures how quickly the nystagmus declines once it has peaked (in seconds): $\text{Fall time}(\mathrm{s})=\text{tlast nystagmus}-\text{tPeak SPV}$.

Duration was the total duration of nystagmus, from the first onset to the cessation of nystagmus (in seconds). Essentially, $\mathrm{Duration}=\mathrm{Latency}+\text{Rise time}+\text{Fall time}$ (if nystagmus occurs), or it can be measured directly as the time between first and last nystagmus beats: $\mathrm{Duration}(\mathrm{s})=\text{tlast nystagmus}-\text{tfirst nystagmus}$.

All features were normalized with reference to the test position.

In a typical BPPV DH-T response, one would expect a short latency (a few seconds), a reasonably high peak SPV, a certain build-up and decay (Rise time and Fall time), and a total duration of less than one minute. In contrast, atypical responses might show differences in one or more of these parameters [8,19]. Features were calculated from the recorded data using custom scripts in MATLAB to ensure consistency and objectivity.

Stage 3: Model Development and Internal Validation

The primary objective was to develop a predictive model that estimates the probability that a given DH-T observation corresponds to typical rather than atypical BPPV. A binary logistic regression model was fitted using typical BPPV as the binary outcome variable (1=typical; 0=atypical), with extracted nystagmus features evaluated as candidate predictor variables. Logistic regression was selected because of its interpretability and suitability for binary classification problems [21].

The model takes the following form [2]: $\operatorname{Logit}(y) = \beta_0 + \beta_1 \times X_1 + \beta_2 \times X_2 + \cdots + \beta_n \times X_n$. Here, each coefficient βₙ represents the change in the log-odds of the case being classified as typical BPPV per unit increase in the respective predictor variable.

Feature selection was guided by clinical relevance and model performance. The final model included latency and peak SPV as predictors. Model coefficients were subsequently incorporated into a clinical implementation script to generate predicted probabilities and binary classifications of typical versus atypical BPPV.

Stage 4: Clinical Implementation

A clinical script (script3’) was developed to load new test data, calculate features, run them through the trained logistic regression, and output a probability and classification (typical/atypical BPPV). The classification was determined based on the threshold set in stage 3. The result supports the diagnostic decision-making process by providing both a probability and a binary outcome.

Stage 5: Validation

Independent test evaluation: After finalizing the model and classification threshold based on the training data and cross-validation, the model was evaluated on an independent test set consisting of four observations. Predicted probabilities and classifications (typical versus atypical BPPV) were generated using the final logistic regression model. The model's classifications were then compared with the expert clinical diagnoses to assess performance on previously unseen data.

Statistical analysis

Statistical analyses were performed in MATLAB (MathWorks, Natick, Massachusetts, United States). Candidate predictor variables included latency, peak SPV, rise time, fall time, and duration. Feature selection was guided by clinical relevance and model performance metrics. Pearson correlation coefficients were calculated to assess multicollinearity among candidate predictors.

Model performance was evaluated using receiver operating characteristic (ROC) curve analysis and area under the ROC curve (AUC). Additional performance metrics included accuracy, sensitivity, specificity, positive predictive value (PPV), negative predictive value (NPV), F1 score, and area under the precision-recall curve (PR-AUC).

To assess model robustness and generalizability, repeated five-fold cross-validation was performed at the patient level using 100 iterations, with each iteration involving a new random partitioning of the dataset. Model performance was evaluated across all validation folds.

The optimal classification threshold was initially identified using Youden's J index and subsequently evaluated using a threshold of 0.5 for clinical implementation [3]. Odds ratios (ORs) and 95% confidence intervals (CIs) were calculated for model coefficients to support clinical interpretability. Statistical significance was defined as p<0.05.

## Results

This section presents the results of a logistic regression model to differentiate between typical and atypical BPPV variants. These findings provide a foundation for assessing the model's potential as a clinical decision support tool to optimize triage and systematic diagnostics.

Study cohort and diagnostic labels

The dataset consisted of 26 patients, 21 women (80.8%) and five men (19.2%), with a mean age of 65±13 years. A total of 133 measurements were recorded, one per eye, during a DH-T. Of these, 128 (96%) were used for training/testing the model, four (3%) were used for final testing, and one (1%) was excluded due to missing data. Sixty-three measurements (47%) were from the right side and 70 (53%) from the left. Diagnostically, 52 (39%) were classified as typical BPPV and 81 (61%) as atypical. 

Nystagmus features in typical and atypical BPPV

**Figure 3:**
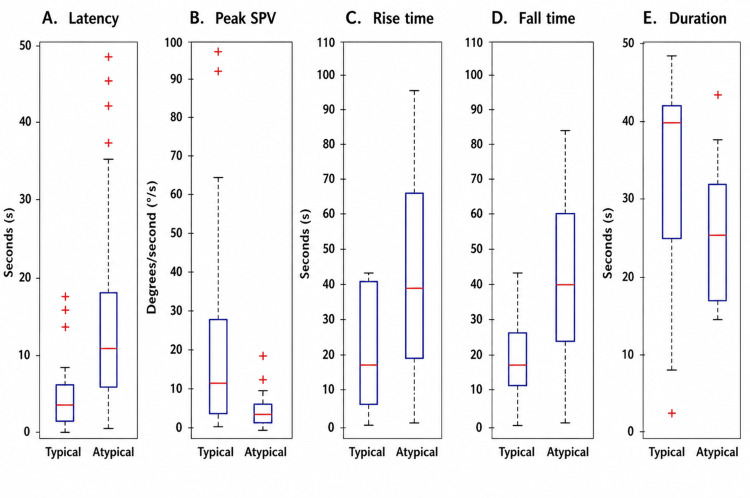
Comparison of nystagmus characteristics between typical and atypical BPPV Boxplots comparing the qualitative nystagmus characteristics between typical and atypical BPPV observations. A shows the latency from the attainment of test position to the onset of nystagmus. B shows the peak SPV. C shows the rise time from nystagmus onset to peak SPV. D shows the fall time from peak SPV to the end of the nystagmus. E shows the total nystagmus duration. Typical BPPV observations demonstrated shorter latency, shorter rise and fall times, and higher peak SPV compared with atypical observations. Boxes represent the IQR, the horizontal line indicates the median, whiskers represent the range of non-outlier observations, and crosses indicate outliers. Typical BPPV recordings demonstrated greater nystagmus intensity than atypical BPPV recordings. Peak SPV was substantially higher in the typical BPPV group, with a median value of 12.59°/s (IQR: 23.19), compared with 3.53°/s (IQR: 2.86) in the atypical BPPV group. Comparisons of latency, rise time, fall time, and duration are shown for both groups. BPPV: benign paroxysmal positional vertigo; SPV: slow-phase velocity; IQR: interquartile range

Quantitative analysis demonstrated clear differences in the temporal and intensity-related features of nystagmus between typical and atypical BPPV presentations. Figure 3 illustrates the median values and variability (IQR) of the five extracted features across both groups.

Latency to Onset

Latency was shorter in the typical BPPV group, with a median of 3.06 seconds (IQR: 3.15), compared to a higher median of 10.60 seconds (IQR: 15.36) in the atypical group. This difference indicates both a longer delay and greater variability in onset for atypical cases.

Peak Slow-Phase Nystagmus Velocity

Typical BPPV observations exhibited higher nystagmus velocities, with a median peak SPV of 12.59°/s (IQR: 23.19), while the atypical group had a much lower median of 3.53°/s (IQR: 2.86). This reflects stronger and more variable nystagmus in typical cases.

Rise Time to Peak

Atypical BPPV cases had a longer rise time, with a median of 26.94 seconds (IQR: 22.46), compared to 17.37 seconds (IQR: 21.96) in the typical group. This suggests a slower build-up of nystagmus intensity in atypical presentations.

Fall Time After Peak

In contrast, typical BPPV showed longer decay phases. The median fall time was 29.37 seconds (IQR: 25.38) in the typical group, versus 15.48 seconds (IQR: 21.19) in the atypical group, indicating that nystagmus tends to persist longer after reaching peak intensity in typical BPPV.

Total Nystagmus Duration

Duration was longest in the typical group, with a median of 47.73 seconds (IQR: 17.29), compared to 36.23 seconds (IQR: 27.06) in the atypical group. This highlights a more sustained nystagmus response in typical BPPV.

Representative nystagmus traces

**Figure 4:**
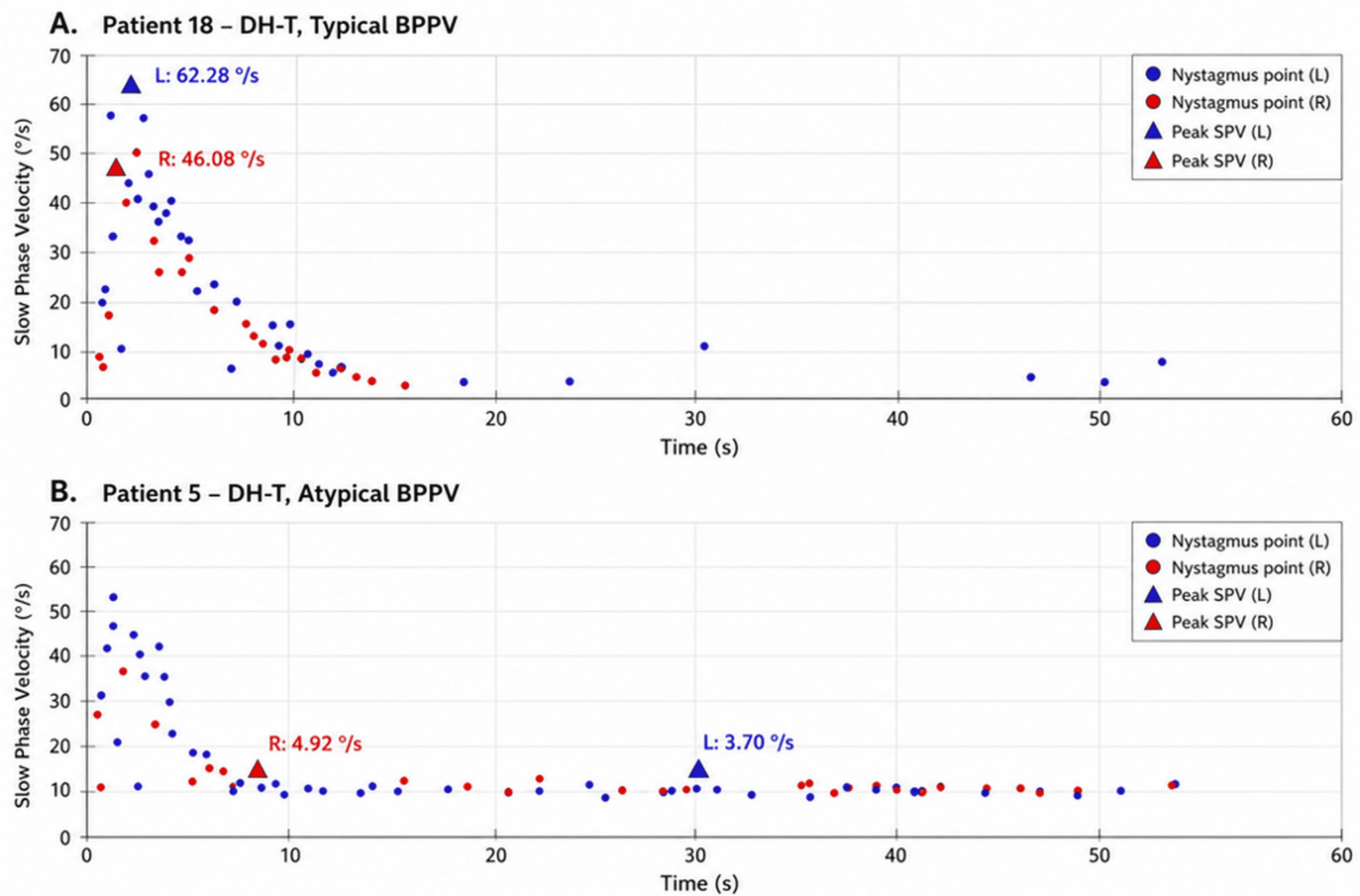
Representative torsional nystagmus recordings during the DH-T maneuver in typical and atypical BPPV Representative examples of torsional nystagmus recordings obtained during the DH-T maneuver. A shows a patient with typical BPPV demonstrating a characteristic transient increase in torsional nystagmus with higher peak SPV values. B shows a patient with atypical BPPV demonstrating lower peak SPV values and a less pronounced nystagmus response. Triangles indicate peak SPV measurements for the left and right eyes. Circular markers represent individual torsional nystagmus measurements recorded throughout the maneuver. Blue markers denote left-eye recordings, and red markers denote right-eye recordings. Time is shown on the x-axis, and SPV is shown on the y-axis. BPPV: benign paroxysmal positional vertigo; DH-T: torsional Dix-Hallpike maneuver; SPV: slow-phase velocity; L: left; R: right

Examples were presented for individual and aggregated nystagmus data. Figure 4 compares the nystagmus data from two cases of BPPV, one typical and one atypical. The top plot shows typical BPPV with strong nystagmus shortly after test positioning, indicating a latency of ~1 s. Peak SPV was 62.28°/s (left eye) and 46.08°/s (right eye), both occurring within the first 1-5 seconds. SPV then declined rapidly, consistent with typical BPPV patterns. The bottom plot shows atypical BPPV. Here, SPV values remained low throughout, with a latency of ~8 s. Peak SPV was 4.92°/s (right eye) and 3.70°/s (left eye), observed at ~8 and 28 s, respectively. The weak and delayed response indicates minimal or absent nystagmus, characteristic of atypical BPPV.

**Figure 5:**
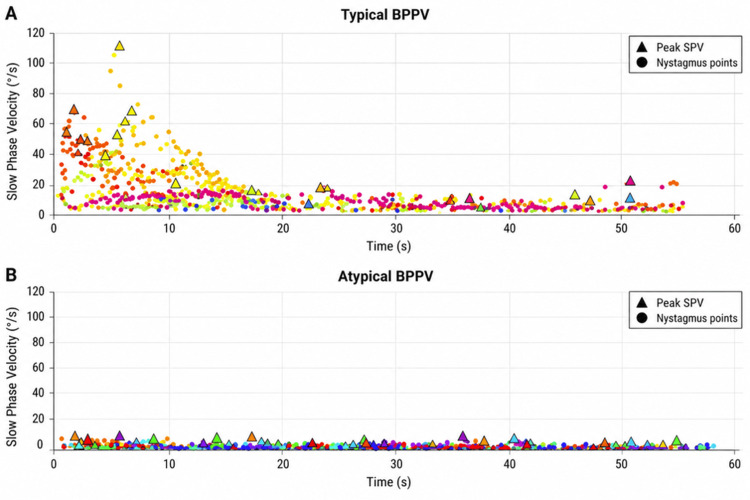
Aggregated torsional nystagmus data during the DH-T maneuver A shows the aggregated torsional nystagmus data from patients with typical BPPV. B shows the aggregated torsional nystagmus data from patients with atypical BPPV. Each circular marker represents an individual SPV measurement plotted over time. Triangles indicate individual peak SPV values for each nystagmus response. BPPV: benign paroxysmal positional vertigo; DH-T: torsional Dix-Hallpike maneuver; SPV: slow-phase velocity

Figure 5 shows the combined torsional nystagmus data for typical and atypical BPPV. The top plot (typical BPPV) reveals a clear pattern with high SPV values early in the test, often peaking above 100°/s within ~10 seconds, followed by a rapid decline. This reflects the typical BPPV response. The bottom plot (atypical BPPV) shows generally lower and more stable SPV values throughout, with no distinct peaks and most values below 10°/s. This indicates a weak or absent nystagmus response, typical of atypical BPPV.

Feature selection

Feature selection evaluated combinations of latency, peak SPV, rise time, fall time, and duration (Table 2) using cross-validation. Peak SPV alone achieved the highest performance (AUC=0.944; accuracy=88.4%; F1=0.834). 

**Table 2:** Classification performance of logistic regression models using torsional nystagmus features Performance metrics for logistic regression models developed using combinations of torsional nystagmus features. Model performance was assessed using AUC, accuracy, and F1 score. AUC: area under the receiver operating characteristic curve; SPV: slow-phase velocity

Features	AUC	Accuracy	F1 score	Data size (n)
Peak SPV	0.944	0.884	0.834	128
Peak SPV + latency	0.936	0.880	0.830	128
Peak SPV + rise time	0.935	0.861	0.814	119
Peak SPV + latency + rise time	0.924	0.866	0.823	119

Table 3 provides the values of the estimated coefficients in the logistic regression. The final logistic regression model used latency and peak SPV due to their clinical and physiological relevance. Peak SPV had a statistically significant positive coefficient (p<0.001), while latency had a negative coefficient. 

**Table 3:** Logistic regression coefficients and odds ratios for the classification of typical and atypical BPPV Parameter estimates from the final logistic regression model used to classify typical and atypical BPPV. OR, 95% CI, and associated p-values are reported for each predictor variable. *Statistically significant parameter estimate (p<0.05). OR: odds ratio; CI: confidence interval; SPV: slow-phase velocity; BPPV: benign paroxysmal positional vertigo

Parameter	Coefficient	OR	95% CI lower	95% CI upper	P-value
Intercept	-3.444	0.03	0.005	0.20	0.0002*
Latency (s)	-0.072	0.93	0.84	1.02	0.14
Peak SPV (°/s)	0.496	1.64	1.30	2.07	0.00003*

Table 4 provides the estimated coefficients in the logistic regression including the intercept, latency, and peak SPV coefficients and OR for each. 

**Table 4:** Model output for independent test measurements used in the final model evaluation Predicted probabilities and classifications generated by the final logistic regression model for the independent test dataset. Model classifications were compared with expert clinical assessment to evaluate agreement between automated and expert classifications. BPPV: benign paroxysmal positional vertigo; SPV: slow-phase velocity

Latency (s)	Peak SPV (°/s)	Probability of typical BPPV (%)	Model assessment	Expert assessment
6.11	12.02	88.68	Typical	Typical
41.95	2.28	0.49	Atypical	Atypical
14.20	1.91	2.88	Atypical	Atypical
26.70	22.42	99.68	Typical	Typical

Threshold selection

**Figure 6:**
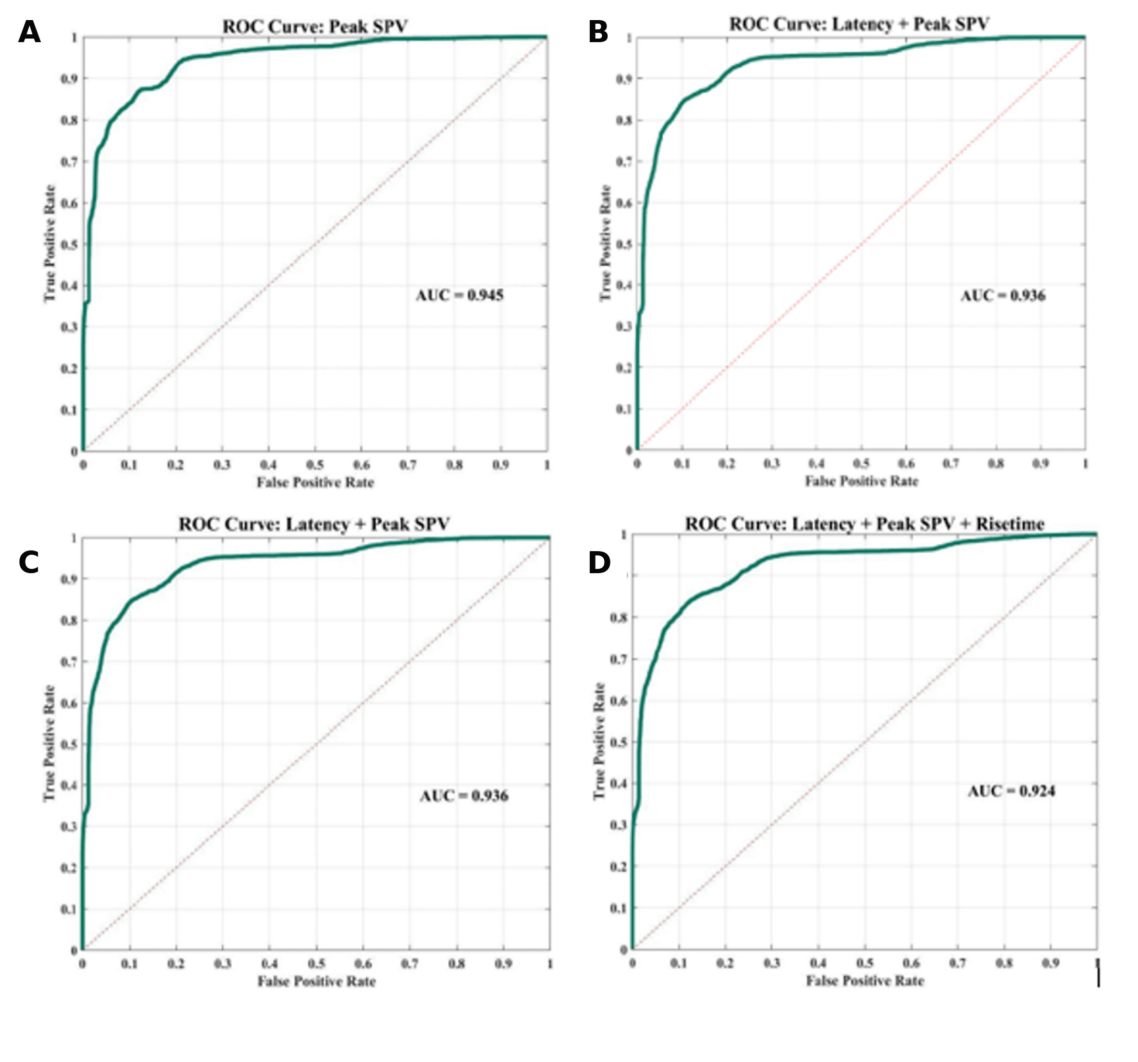
ROC curve analysis of logistic regression models for the classification of typical and atypical BPPV ROC curves demonstrating the performance of logistic regression models for differentiating typical and atypical BPPV using progressively combined nystagmus features. A shows the classification performance using peak SPV alone. B shows the model incorporating latency and peak SPV. C shows the model incorporating latency, peak SPV, and duration. D shows the model incorporating latency, peak SPV, and rise time. The diagonal dashed line represents the line of no discrimination. AUC values are shown within each panel and indicate the overall classification performance of each model. ROC: receiver operating characteristic; AUC: area under the ROC curve; BPPV: benign paroxysmal positional vertigo; SPV: slow-phase velocity

Figure 6 shows the ROC curves for peak SPV and latency, illustrating the relationship between the true-positive rate and false-positive rate across a range of thresholds. ROC curve analysis yielded an AUC of 0.936, indicating excellent discriminatory performance. Based on Youden's J index, the optimal threshold was identified at 0.4 (Youden's J=0.743), corresponding to a sensitivity of 84.5%, a specificity of 89.9%, an accuracy of 87.8%, and an F1 score of 0.839. However, the final classification threshold was not selected solely based on Youden's J index. A threshold of 0.5 was used in subsequent analyses to ensure a more balanced trade-off between sensitivity and specificity and to align with conventional probabilistic interpretation and clinical decision-making practice.

Precision-recall (PR)

PR-AUC was 0.857, indicating strong precision and recall. Lower thresholds improved sensitivity, while higher thresholds improved precision. The model consistently outperformed baseline classification.

Repeated k-fold cross-validation

A repeated five-fold cross-validation with 100 repeats yielded an AUC of 0.936±0.007, an accuracy of 88.0±0.9%, a sensitivity of 78.2±1.9%, a specificity of 93.9±1.1%, and an F1 score of 0.830±0.014.

Independent test evaluation

Model classifications were concordant with expert assessment for all four test observations (Table 4). The test included two typical and two atypical cases, where typical cases showed short latency and high SPV, while atypical cases demonstrated long latency and low SPV.

A logistic regression model that differentiates between typical and atypical BPPV using VNG data from the DH-T may have clinical value. The model demonstrated strong discriminatory performance in internal validation, with AUC values up to 0.944 (see Table 3). However, although performance was also evaluated on a held-out test set, this dataset was small and originated from the same participant cohort as the training data, limiting the degree of independence. Consequently, while the results are promising, further validation in larger and more diverse patient populations is required to assess the model's generalizability in real-world clinical settings.

## Discussion

This study developed a logistic regression-based model to differentiate typical from atypical BPPV using objective VNG measurements, following established principles for clinical prediction model development [22]. The model was designed to enhance diagnostic precision and support clinical decision-making. The study cohort was predominantly female (80.8%), consistent with established BPPV epidemiology, where women demonstrate higher prevalence and report more severe symptoms [23] although this imbalance may limit generalizability to male populations. The mean age of 65±13 years reflects the age-related increase in BPPV prevalence; however, the wide age range introduces variability, as nystagmus characteristics may be influenced by age independently of BPPV subtype [10].

The relatively high proportion of atypical BPPV cases (61%) likely reflects referral bias associated with specialized vestibular clinics and may not fully represent typical BPPV presentations encountered in general practice [22,24]. In addition, previous studies have shown that asymptomatic individuals may exhibit nystagmus patterns resembling BPPV, potentially leading to false-positive classifications when diagnosis relies solely on objective eye movement data [10].

Interpretation of key parameters

Typical BPPV exhibited shorter latency (median: 3.06 s) and higher peak SPV (median: 12.59°/s), consistent with posterior canal BPPV pathophysiology, where free-floating otoconia provoke a rapid vestibular ocular reflex (VOR) response. Atypical BPPV demonstrated longer latency (median: 10.60 s), lower peak SPV (3.53°/s), and slower rise time [19]. Typical BPPV also showed longer fall time and overall nystagmus duration, which may reflect vestibular adaptation following otoconia movement [24].

Feature selection and model development

The final model included two predictors: peak SPV and latency. The model achieved an AUC of 0.936 and an F1 score of 0.830. Peak SPV alone demonstrated high discriminatory power (AUC=0.944), while latency was retained for physiological relevance. The low negative correlation between features (r=-0.313) indicates that they capture distinct aspects of the nystagmus response rather than redundant information [25]. Including latency therefore supports a more physiologically grounded model without introducing substantial collinearity or overfitting. Additional features, such as rise time and duration, were not included due to limited evidence supporting their clinical utility [25]. However, this assumption and initial findings need to be validated in a larger heterogeneous sample. More complex models with additional features were avoided due to a lack of supporting evidence for the clinical use of variables like rise time and duration.

Threshold selection and performance

Cross-validation indicated that a lower threshold of 0.4 increased sensitivity (84.5%) but reduced specificity (89.9%). A threshold of 0.5 was selected for subsequent analyses, resulting in a sensitivity of 78.2% and a specificity of 93.9%. High specificity was prioritized to reduce the risk of misclassifying atypical cases as typical [22,26]. Evaluation on four previously unseen observations yielded correct classifications for all cases, although the small test set limits broader generalizability.

Clinical implementation and prototype

A conceptual prototype was developed to present model outputs in an interactive, clinician-friendly format. By visualizing both probability and raw data, the prototype aims to support decision-making, especially in complex BPPV cases. Future steps include usability testing and stakeholder feedback to refine the interface and assess practical value.

Methodological considerations

A retrospective, cross-sectional design was used to align model development with clinical practice. Real-world clinical data strengthens internal validity, but subjective data processing by a single expert introduces bias [27]. Manually edited data files carry risks of information bias, though automated deep learning methods may offer more reliable alternatives in the future. Repeated measures from the same patients were treated as partly independent due to possible otoconia displacement over time. Nevertheless, the lack of independence can lead to overfitting, emphasizing the need for cautious interpretation [28].

Logistic regression was selected due to its interpretability and transparency, which are important considerations for clinical prediction models where understanding the relationship between predictors and outcomes is essential for clinical trust and implementation. In addition, the relatively limited cohort size in the present study motivated the use of a parsimonious modeling approach that is less prone to overfitting compared with more flexible machine learning methods. While more advanced nonlinear models could potentially capture complex relationships in the data and achieve improved predictive performance, such models often require substantially larger datasets to ensure stable training and generalizable results. In smaller cohorts, highly flexible models may fit noise rather than underlying signal, thereby reducing their reliability in clinical applications. Nevertheless, alternative classical machine learning approaches, such as k-nearest neighbors, random forests, or support vector machines, may offer improved performance in higher-dimensional feature spaces and could be considered in future investigations [29].

The growing availability of model interpretation frameworks, such as SHAP (SHapley Additive exPlanations) [30], may help bridge the gap between predictive performance and interpretability when applying more complex models. Future studies using larger and more heterogeneous cohorts could therefore explore these approaches to better characterize the potential upper bound of predictive performance while maintaining clinically meaningful interpretability. Given the scope of the present study as an initial model development project, we did not perform extensive subgroup analyses or external validations which leads to future work in gaining a better understanding of how to predict posterior canal BPPV.

Limitations

This study has several limitations. The dataset was relatively small, consisting of 26 patients and 133 test observations, with repeated and bilateral measurements increasing the total observations. The limited number of unique patients may restrict generalizability across clinical settings or populations. The cohort composition may not reflect typical prevalence distributions, as the proportion of atypical BPPV cases (61%) likely reflects referral bias at specialized clinics. Metrics such as AUC are robust to class imbalance, but prevalence-dependent measures, including PPV, may differ in other populations.

BPPV classification was based on expert clinical judgment, which, while treated as the reference standard, is inherently subjective and may introduce labeling bias. The model was internally validated with repeated cross-validation and externally tested on a small independent set of four cases. Although agreement with expert assessment was high, this limited external evaluation does not replace validation in larger, prospective, and more heterogeneous cohorts. The small sample size used for independent validation limits the ability to draw firm conclusions regarding generalizability. Further validation in larger and more diverse independent datasets is therefore necessary to confirm the robustness and clinical applicability of the model.

The model was restricted to two features, latency and peak torsional SPV, selected for physiological relevance and discriminatory performance. Additional parameters, such as horizontal and vertical nystagmus, duration, rise time, and fall time, may provide complementary information and should be investigated in future studies to enhance robustness and generalizability.

Given the exploratory nature of this study, individual VNG measurements were treated as independent observations; however, repeated measurements from the same participant may introduce within-subject correlation. To mitigate this, cross-validation was performed at the patient level, ensuring that data from the same participant did not appear simultaneously in both training and test sets. Future studies with larger datasets should further address clustering effects using mixed-effects modelling or other subject-level validation strategies.

Future work should focus on validating the model in larger and more diverse populations and integrating it into broader diagnostic workflows. Diagnostic labels should ideally be confirmed through expert consensus, such as a Delphi process. Extension of the model to differentiate other vestibular conditions, including Ménière's disease, vestibular migraine, and additional BPPV subtypes, will require larger datasets and the incorporation of horizontal, vertical, and torsional eye movement features. Expansion of the model to the identification of horizontal canal BPPV and to distinguish between typical posterior/horizontal canal BPPV and other rare forms of BPPV, central conditions, and vestibular migraine is necessary.

## Conclusions

This study demonstrates that a logistic regression model using latency and peak SPV of torsional nystagmus can reliably differentiate typical from atypical BPPV. The model provides an interpretable, clinically applicable tool that supports improved diagnostic accuracy and informed treatment decisions for patients with positional vertigo. These results establish a foundation for further validation in larger and more diverse populations and for future expansion to other vestibular disorders.

In contrast to prior deep learning approaches that primarily detect the presence of nystagmus, the present study focuses on extracting clinically interpretable temporal parameters from VNG recordings during the Dix-Hallpike maneuver and evaluating their diagnostic value using an explainable statistical model.
